# Design, inverted vat photopolymerization 3D printing, and initial characterization of a miniature force sensor for localized in vivo tissue measurements

**DOI:** 10.1186/s41205-021-00128-2

**Published:** 2022-01-04

**Authors:** Shashank S. Kumat, Panos S. Shiakolas

**Affiliations:** Mechanical and Aerospace Engineering Department, The University of Texas at Arlington, S Nedderman Dr, Arlington, 76019 TX USA

**Keywords:** Micro-force sensor, Human confined space, Tissue evaluation, 3D printing, Design, Sensor performance characterization, In vivo measurements

## Abstract

**Background:**

Tissue healthiness could be assessed by evaluating its viscoelastic properties through localized contact reaction force measurements to obtain quantitative time history information. To evaluate these properties for hard to reach and confined areas of the human body, miniature force sensors with size constraints and appropriate load capabilities are needed. This research article reports on the design, fabrication, integration, characterization, and in vivo experimentation of a uniaxial miniature force sensor on a human forearm.

**Methods:**

The strain gauge based sensor components were designed to meet dimensional constraints (diameter ≤3.5*mm*), safety factor (≥3) and performance specifications (maximum applied load, resolution, sensitivity, and accuracy). The sensing element was fabricated using traditional machining. Inverted vat photopolymerization technology was used to prototype complex components on a Form3 printer; micro-component orientation for fabrication challenges were overcome through experimentation. The sensor performance was characterized using dead weights and a LabVIEW based custom developed data acquisition system. The operational performance was evaluated by in vivo measurements on a human forearm; the relaxation data were used to calculate the Voigt model viscoelastic coefficient.

**Results:**

The three dimensional (3D) printed components exhibited good dimensional accuracy (maximum deviation of 183*μm*). The assembled sensor exhibited linear behavior (regression coefficient of *R*^2^=0.999) and met desired performance specifications of 3.4 safety factor, 1.2*N* load capacity, 18*mN* resolution, and 3.13*%* accuracy. The in vivo experimentally obtained relaxation data were analyzed using the Voigt model yielding a viscoelastic coefficient *τ*=12.38*sec* and a curve-fit regression coefficient of *R*^2^=0.992.

**Conclusions:**

This research presented the successful design, use of 3D printing for component fabrication, integration, characterization, and analysis of initial in vivo collected measurements with excellent performance for a miniature force sensor for the assessment of tissue viscoelastic properties. Through this research certain limitations were identified, however the initial sensor performance was promising and encouraging to continue the work to improve the sensor. This micro-force sensor could be used to obtain tissue quantitative data to assess tissue healthiness for medical care over extended time periods.

## Background

Urinary bladder cancer is considered as a leading cause of patient mortality among cancers, with 83,730 estimated new cases in 2021, corresponding to 4.4*%* of all new cancer cases in the US alone [1]. Tests performed for bladder cancer diagnosis include physical exam and health history, endoscopy, urinalysis, urine cytology, cystoscopy, intravenous pyelogram (IVP) and biopsy [2]. Diagnostic tests such as physical exam, internal exam urine cytology and cystoscopy cannot be used to quantitatively identify localised in vivo tissue viscoelastic properties. Some of the current technologies employed to interact with tissue in confined spaces include endoscopy [3], cystoscopy [4, 5], prolapse assessment [6, 7], biopsy [8], and analysis of the exterior bladder wall movement [9].

Urinary incontinence (UI) is another medical condition affecting 33 million Americans classified as either stress urinary incontinence or urge urinary incontinence [10]. *Ex vivo* tensile testing demonstrated that the tissue stiffness index helps to identify the severity of pelvic floor disorder and UI [11, 12].

Studies showed that a tumorous surface exhibits a higher stiffness compared to healthier surrounding tissues [13–15]. As such, tumor mechanics significantly differ from that of normal tissue. Hence, identifying localized viscoelastic properties of tissues can be advantageous in assessing the healthiness of organs [6–8, 16, 17].

Instrumentation used in clinical settings limits the physicians ability to deliver consistent care [18, 19]. Therefore, there is a need for diagnostic tools capable of accessing confined spaces in the body to interact with the tissue at the local level and then use interaction results to evaluate quantifiable tissue properties for medical diagnosis and care. The quantification of vaginal tissue alterations could aid in detecting women at risk of pelvic organ prolapse (POP) at an early stage [17].

A study conducted by *Abraham* showed that pelvic organs can undergo a reaction force of approximately 1.2*N* with an indentation depth ranging from 8−10*mm* [6]. Monitoring of tissue viscoelastic properties over a time period could lead to the development of better treatments and outcomes for women suffering from POP [17]. Therefore, evaluation of localized tissue properties will be essential for disease prevention and/or detection. Being able to monitor the applied force and acquire reaction force information could result in improved diagnostics [3, 6, 20]. Therefore, there has been interest in the development of micro-force sensors that could be attached at the end of diagnostic instruments to interact with the tissue.

A micro-force sensor with diameter less than 3*mm* and a total length of 15*mm* capable of sensing an axial load in the range 0−4*N* with a resolution of 14*mN* was reported by *Deng et. al* [20]. Conventional manufacturing processes including laser machining were used for prototyping a Nitinol alloy based force sensor and for prototyping subsequent design revisions aiming to simplify their sensor design. Their design suffered from incorporating necessary complex features due to limitations of conventional manufacturing processes. A triaxial force sensor with a diameter of 4*mm* designed by Li et. al. was placed at the tip of a catheter for measuring the interaction force between tissues [21]. This sensor had an accuracy and resolution of 2.7*mN* and 0.6*mN* respectively in the axial direction and capable of withstanding an axial load of 0.8*N* [21]. Components of the force sensor by Li et. al. were fabricated using 3D printing technology due to the presence of complex features [21]. The sensors investigated by Deng et. al. and Li et. al. were based upon the working principle of fiber optics [20–22]. Fiber brag grating (FBG) sensing technology can be used as a strain measurement sensor by measuring the change in period of the wavelength of emitted light source. The use of FBG limits the ability of the sensor holding equipment to attain large bending angles (∼180^*o*^) and small bending radius in confined spaces such as the bladder [21, 23]. In addition, FBG instrumentation is bulky and costly which could further limit widespread use of FBG based diagnostic devices [23, 24]. The limitations of FBG based sensors could be overcome by strain gauge based force sensors. A strain gauge based force sensor is a well developed and mature technology and its working principle relies on measuring the relative change in resistance due to relative changes in developed strain caused by generated reaction forces upon engagement with tissue.

Rapid prototyping through 3D printing can significantly improve not only the product development life-cycle and design process by quickly prototyping design iterations but also address the long lead times required to develop the tools and fixtures necessary to fabricate components using traditional machining [25]. 3D printing has gained popularity due to its advantages such as ability to fabricate geometrically complex features, availability of variety of bio-materials, and ability to fabricate personalized devices that are safe and cost effective [26–28].

In this work, it is proposed to research the design, fabrication and characterization of an encapsulated uniaxial micro-force sensor less than 3.5*mm* in overall diameter. This force sensor is envisioned to be attached on the tip of a micro-robot capable of positioning and properly orienting the sensor in confined and hard to access areas of the body. The sensor will probe the tissue by controlled indentation and record the reaction and relaxation forces as function of time. The measured data will then be used to evaluate localized biomechanical properties of the tissue [29, 30].

In order to address the challenges faced by conventional manufacturing processes and expedite the fabrication of design iterations for this research, the components of the micro-force sensor were fabricated using 3D printing technology. One of the major challenges in fabricating the proposed micro-force sensor using 3D printing was to meet geometric constraints while maintaining the desired performance specifications.

The operational and design requirements for the sensor will be introduced. Procedures followed for the design and fabrication of the sensing element will be discussed using traditional machining. The fabrication of various sensor components using 3D printing along with advantages and challenges faced using a Form3 printer will be discussed. The procedures followed and tools developed to characterize the performance of the assembled sensor will be presented. Subsequently, the development and use of a testbed to evaluate the in vivo operational performance of the sensor when interacting with a human forearm and the use of force relaxation data to evaluate biomechanical properties (viscoelastic constant) according to the Voigt model will be presented. The manuscript will close with a discussion on the next steps to address limitations identified through this research study.

## Methods

### Design requirements

In vivo measurements for evaluating viscoelastic properties of organs such as the bladder or pelvic organ tissue are beneficial in that they provide for the quantitative assessment of the healthiness of the tissue. The design requirements of the micro-force sensor proposed in this research must consider accessibility to the confined space site as it relates to its size, the range of the normal force to be applied at the tip of the sensor and operational environment conditions.

According to the study conducted by Hudson et al., ideally an outside diameter of a flexible endoscope must be ≤7.4*Fr* (2.4*mm*) to avoid ureteric dilatation [31]. In order to maintain a balance between the passage and durability of the device, the outer diameter of commonly used flexible endoscopes in adults falls within a range of 15*Fr* to 25*Fr* (5.0*mm* to 8.3*mm*) [5]. The force sensor designed by Li. et. al. for accessibility in confined space was reported to be 4*mm* in diameter [21]. *Goud* suggested that a device with a limited contact duration (≤24*h*), needs to be fully packaged and sterilizable to maintain biocompatibility [32].

The environmental constraints include accessibility to confined spaces in the human body, ability to operate in wet/moist conditions and biocompatibility. The issues associated with biocompatibility and the moist operating environment of the bladder could be addressed by encapsulating the sensor with a biocompatible protective sheath or covering. One of the major constraints for the proposed force sensor considering the intended application of bladder diagnosis was the sensor outside diameter which was preferred to be (≤5*mm*) [5].

Studies showed that pelvic organs can undergo a reaction load of approximately 0.8−1.2*N* with indentation depth ranging from 8−10*mm* [6, 7, 17]. The proposed micro-force sensor must safely withstand a normal load of 1.2*N* with a safety factor suitable for medical devices; in our research we aim for a safety factor greater than 3 similar to that used by Deng et. al. who used a safety factor of 3 [20]. The design requirements for the proposed micro-force sensor are summarized in Table 1.

**Table 1:** Design requirements for the proposed micro-force sensor

Characteristics	Specifications
Dimensions	Sensor housing diameter ≤3.5*mm*
Force Range	Normal force ≤1.2*N*
Resolution	20*mN*
Safety Factor	≥3
Operating environment	Wet/Moist
Others	Biocompatibility

### Overall sensor concept and working principle

**Fig. 1:**
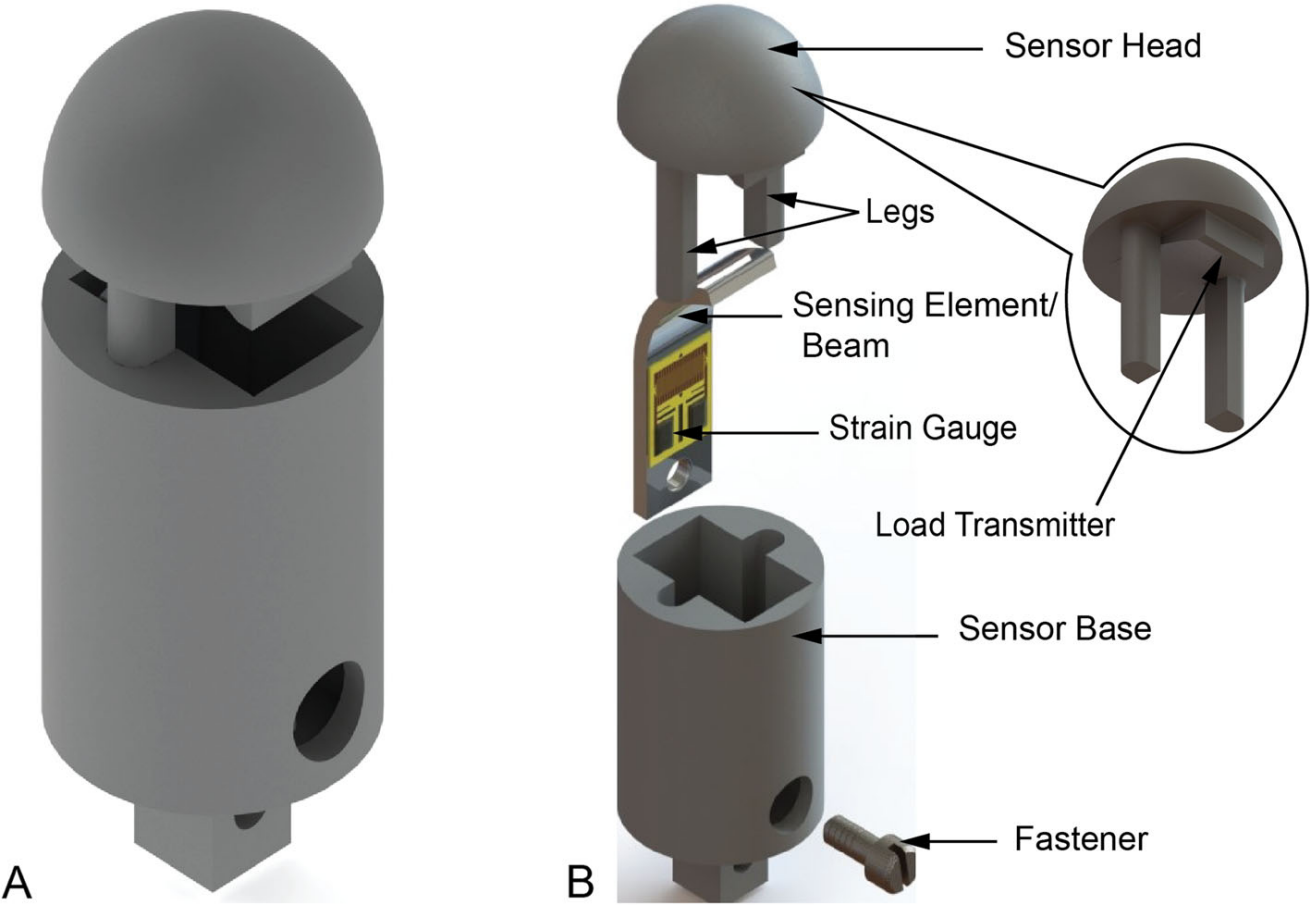
Solid models of proposed sensor; overall and exploded view, **A** Assembled sensor model, **B** Exploded view of the sensor model

The CAD model of the proposed sensor is presented in Fig. 1; Fig. 1a is the assembled view and Fig. 1b is the exploded view with an inset showing features of the sensor head indiscernible from the exploded view. The biocompatible covering is not shown for visualization purposes. The nomenclature used for the sensor components follows the labels in Fig. 1b.

The sensor will be positioned at the point of interest and oriented to be perpendicular to the tissue to be queried. The hemispherical surface of the sensor head will engage with the tissue. When the sensor head comes in contact with and indents the tissue, a reaction force will be generated on the hemispherical surface of the sensor head and transferred to the sensing element/beam via the load transmitter on the sensor head. The legs of the sensor head will ensure a sliding motion relative to the sensor base. For an in vivo diagnostic operation, the sensor base could be attached on a micro-robot to gain access to the confined space [29, 30]. The deformation of the beam due to the applied load from the sensor head will generate strain in the beam (the other end of the beam is fastened to the sensor base). The fastener used to affix the beam to the sensor base was a standard 0.5*mm* Unified National Miniature (UNM) fillister head screw by Antrin Miniature Specialties Inc. (Fallbrook, CA) [33]. The sensing element design considered the allowable space in the sensor base cavity, while maximising the strain sensed without undergoing plastic deformation.

This sensing element served as the mounting structure for a metal foil strain gauge (*N*2*K*−06−*S*5024*G*−50*C*/*DG*/*E*3) by Micro-Measurements (Wendell, NC) with planar dimensions 1.9*mm*×1.4*mm* [34].

**Fig. 2:**
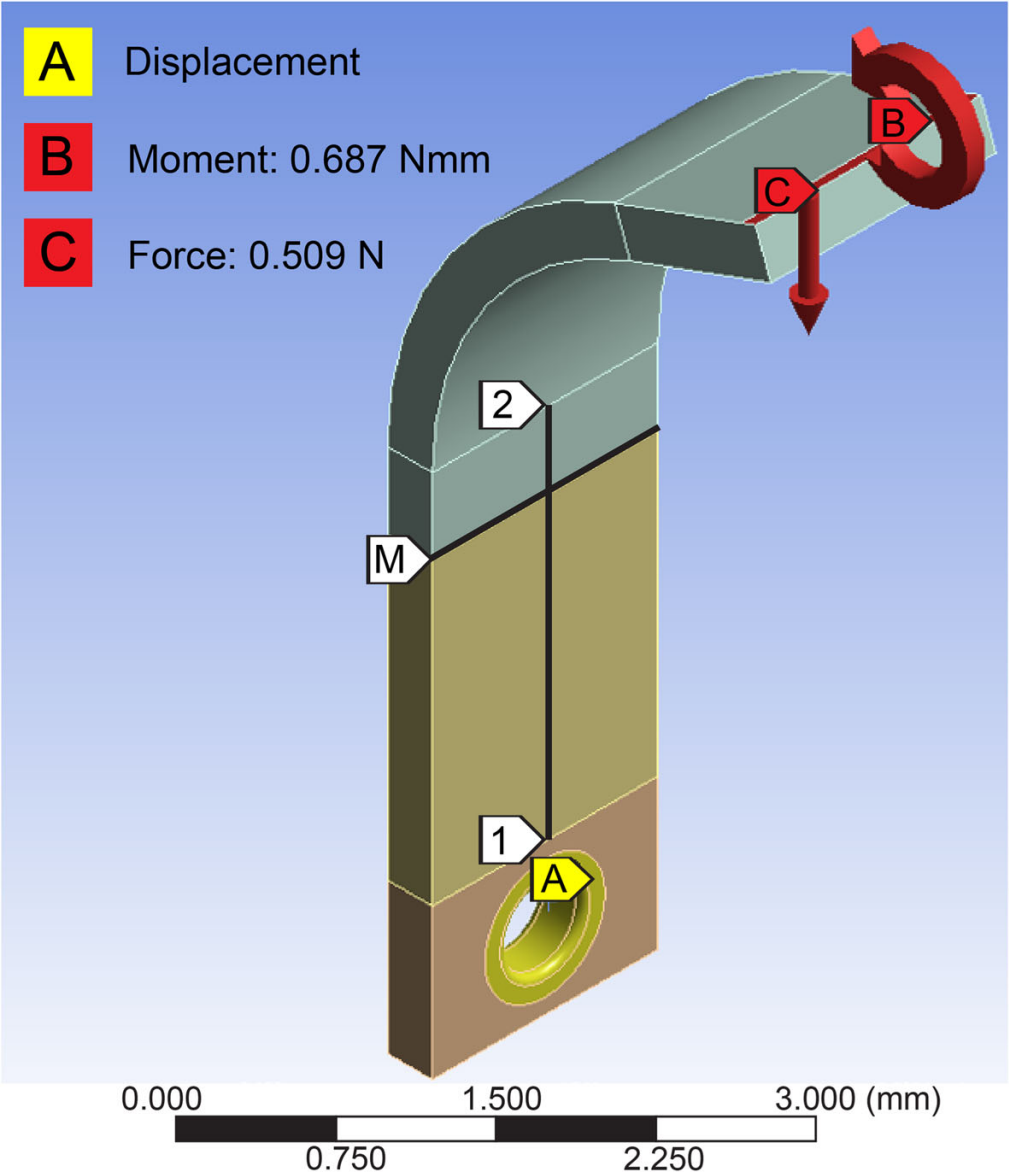
Sensor element FE model

Finite element (FE) analysis was performed to identify the dimensions of the sensing element while maximizing the strain experienced due to applied load while remaining in the elastic region and meeting available space constraints. Figure 2 shows the sensing element FE model with applied loads and boundary conditions. As shown in Fig. 1b, the load transmitter does not engage with the sensing element (beam) at the free end but rather on the curved part of the beam at a distance from the free end. This loading condition was modeled as an equivalent normal load and moment at the free end of the beam for the FE analysis. A zero displacement was defined at location *A* where the fastener will fix the beam element on the sensor base component. Location *M* defines the center of the active area of the strain gauge. The path from point 1 to point 2 (Fig. 2) on the surface of the sensing element represents the path along which the strain will be evaluated since the strain gauge will be attached along this path. The Von-Mises stress, defined as the uniaxial tensile stress due to the distortion energy by actual combination of applied stresses was used as the failure criterion for designing components within the defined safety factor [35].

### Sensor component fabrication

The size and complex features of the sensor head and sensor base components could not be easily fabricated using traditional machining processes. This created an impediment to prototyping several iterations of the sensor during design improvements and we investigated the use of 3D printing technology for fabrication.

3D printing served as the rapid prototyping platform for the proposed sensor design due to the geometric features and size of its components without the need to fabricate custom fixtures and molds for traditional machining processes. 3D printing was also used to fabricate the fixtures needed for sensor characterization and experimentation. *Fateri & Gebhardt* discussed pros and cons of five 3D printing processes; Stereolithography (SLA), Selective Laser Sintering (SLS), Fused Deposition Modeling (FDM), Powder-Binding Bonding (3DP) and Layer Laminate Manufacturing (LLM) [36].

As discussed by Ravi et. al., the mean dimensional error for complex geometric models of human organs fabricated using Form3B VP printer (Formlabs, Somerville, MA, USA) with a commercially available Grey material was 260*μm* with good surface quality [37].

The fabrication specifications using a Form3 printer were well within the required feature size of the sensor housing components [38]. Low Force Stereolithography (LFS), the fabrication process selected for prototyping the sensor components, is the 3D printing technology of the Form3 printer by Formlabs using Formlabs Grey material [39]. The Grey material could be used to fabricate structures with a layer thickness measuring 25*μm* as opposed to 50*μm* and 100*μm* with other Form3 compatible materials [39]. The fabrication slicing paths were generated using the PreForm software by Formlabs. The sensor components were fabricated at the MAE Design Innovation laboratory (The University of Texas at Arlington, TX).

### Sensor characterization

**Fig. 3:**
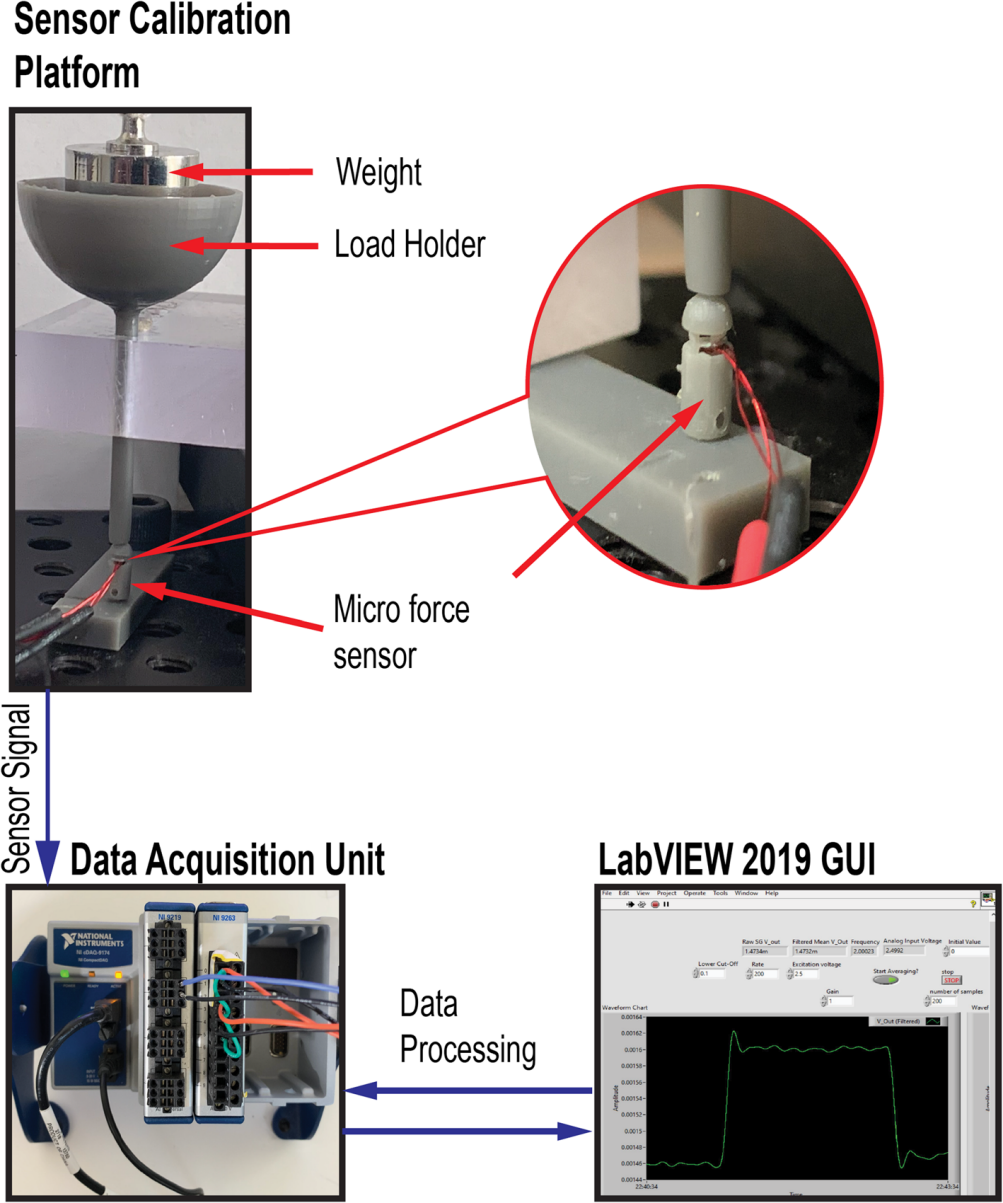
Data acquisition system for sensor calibration setup

This section describes the experimental setup developed to characterize the performance of the prototyped sensor. The characterization experiments were conducted using randomized experiments (to prevent biasing the results) with one factor (applied load) at five different levels. These experiments were designed to evaluate precision, sensitivity, resolution and accuracy of the micro-force sensor as well as its calibration equation. The assembled sensor was set up on the calibration test platform, as shown in the upper left hand corner of Fig. 3.

The calibration test platform shows a load holder which apply the load in the normal direction on the sensor head. A set of five dead weights were used for the calibration experiments of the micro-force sensor. The applied load would cause the beam and attached strain gauge to deform. The deformation in the strain gauge would generate a signal which was read by a data acquisition (DAQ) unit, a 24-bit NI-9219 (National Instruments Inc. Austin, TX) module, shown in the left hand bottom corner of Fig. 3. The NI-9174 chassis houses the NI-9219 module and was USB connected to a computer running LabVIEW by National Instruments. A LabVIEW graphical user interface was developed to display and record the raw data acquired by the DAQ system. The resistance from the strain gauge was recorded with just the sensor head resting on top of the sensing element and found to be 5014.89*Ω*. This resistance will be referred to as the nominal or no-load resistance of the attached strain gauge. The application of a load resulted in a change in the resistance where a decrease in the resistance value indicated a compression load. The change in resistance, *ΔR*_*sg*_, nominal resistance, *R*_*sg*_, and gauge factor, *K*, of the strain gauge were used to evaluate the equivalent strain due to an applied load according to Eq. 1.

1 $$ \epsilon=\frac{1}{K} \frac{\Delta{R_{sg}}}{R_{sg}}   $$

The calibration equation relating the measured strain, *ε*, (strain experienced by the strain gauge) to the applied load, *F*, and the calibration factor, *C*_*f*_, is presented in Eq. 2.

2 $$  \epsilon = C_{f} \times F  $$

The equivalent sensed load using strain measurements during the tissue properties experimentation was evaluated by re-arranging Eq. 2 to yield Eq. 3.

3 $$  F =\frac{\epsilon}{C_{f}}  $$

The resolution of a sensor is defined as the smallest absolute change in resistance that could be detected by the measurement device [40]. Sensitivity is the ability of the sensor to capture the smallest change in output variable (resistance) for a given input variable (applied load) [40]. Accuracy of the sensor is defined as the deviation of the measured quantity from the theoretically estimated value [40]. The accuracy of the sensor is evaluated by comparing the strain evaluated from the measured resistance to the theoretical strain obtained from FE analysis. The error between the theoretical and measured strains is evaluated according to Eq. 4.

4 $$ Error \%\,=\, \left \lvert \frac{\text{Theoretical strain} \,-\, \text{Experimental strain}}{\text{Theoretical strain}} \right\lvert \!\times \!100\%   $$

Precision refers to how closely individual measurements are in agreement with each other for a particular loading condition [40]. Precision is computed according to Eq. 5, where *M*_*sd*_ is the maximum deviation observed throughout the measurement and *Avg*(*M*_*sd*_) is the average measurement throughout the five sets of data for the particular loading condition [40].

5 $$  Precision \%=\left (1-\left \lvert{\frac{M_{sd}}{Avg(M_{sd})}}\right \rvert\right)\times 100  $$

### Sensor operational performance

**Fig. 4:**
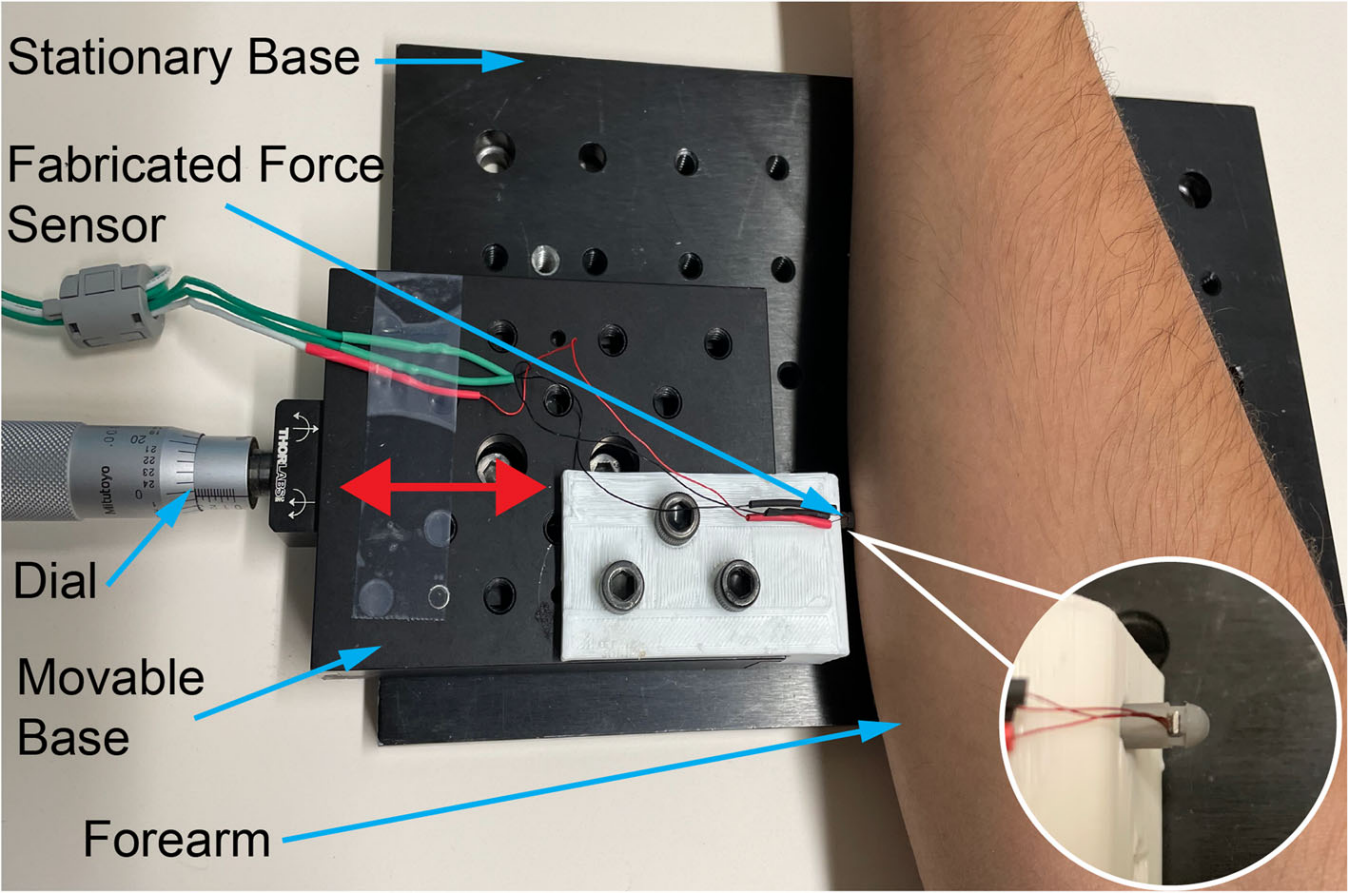
Testbed for in vivo performance evaluation

The testbed shown in Fig. 4 was developed to obtain initial reaction strain data to validate sensor operational performance by measuring tissue relaxation, and then use the relaxation data to quantitatively characterize biomechanical properties of the interrogating tissue on a human forearm.

The testbed allowed for the sensor to be manually translated using a micrometer dial to a desired indentation distance. After indentation, the sensor was kept at this position for a predefined time while the tissue relaxed. The collected strain data as function of time were transformed into force using the developed characterization Eq. (3). A number of different models have been proposed to evaluate biomechanical tissue properties such as Voigt model, Kelvin-Voigt model, Prony series, and Neo-Hookean [7, 41–44]. In this research, the transformed relaxation force data was used to identify viscoelastic properties of the tissue as function of the relaxation time according to the Voigt model [6, 7, 17]. The Voigt model quantifies the ratio of the elastic constant to the damping coefficient as a function of time and quantification of this ratio helps to estimate viscoelastic property of the tissue [7]. The solution to the Voigt model is given by Eq. 6 where *f*(*t*) is the measured reaction force response during tissue relaxation, *f*_*peak*_ is the peak reaction load sensed by the sensor, *f*_*residual*_ is the residual force, and *τ* is a coefficient representing the tissue recoil during the recovery phase [7].

6 $$ f(t) = (f_{peak}-f_{residual}) e^{-t/\tau} + f_{residual}   $$

The initial tissue characterization using the fabricated force sensor demonstrated promising results.

## Results

In this section, the results of the analyses performed to design and fabricate the sensor will be presented. Also, lessons learnt using the Form3 3D printer will be discussed. The fabricated and assembled sensor characterization performance matrix will be presented. Finally, the results of the in vivo forearm tissue characterization experiment will be presented.

The FE analysis results showed that the beam element experienced a Von-Mises stress of 35.9*MPa* and 81.9*MPa* near the mounting hole when loads equivalent to 510*mN* and 1.2*N* were applied respectively. A factor of safety of 3.4 was evaluated for the desired load capacity of 1.2*N* using the Von-Mises stress criterion.

**Fig. 5:**
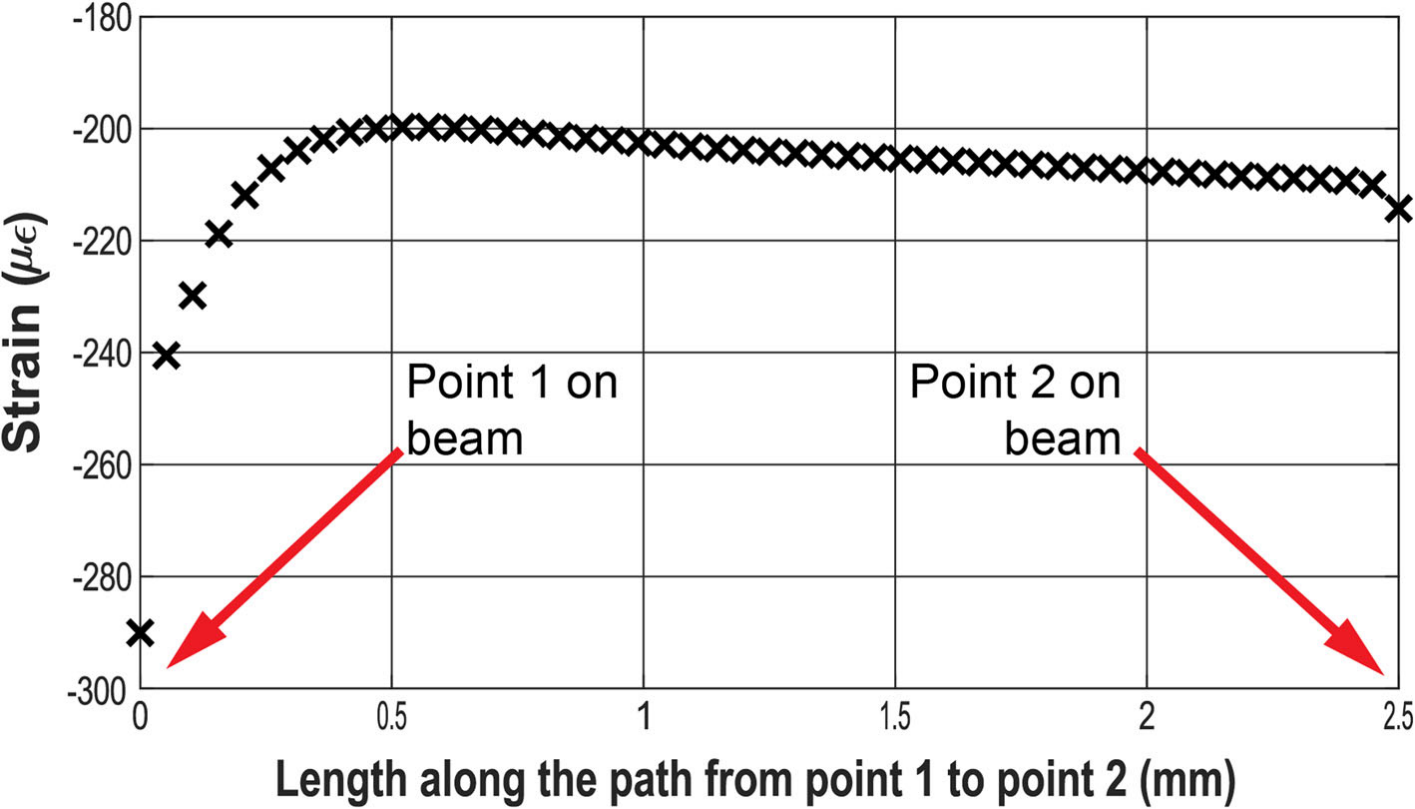
Theoretical strain evaluated along the path from point 1 to point 2 on the surface of the beam due to an applied load equivalent to 510*mN*

The strain experienced on the path between points 1 and 2 of the beam due to an applied load equivalent to 510*mN* is presented in Fig. 5. The strain exhibited a linear behavior along a section of the path indicating that the active length of the strain gauge needed to be placed in this region. The 0 and 2.5 (units) on the horizontal axis represent points 1 and 2 on the sensing element respectively (see Fig. 2).

**Fig. 6:**
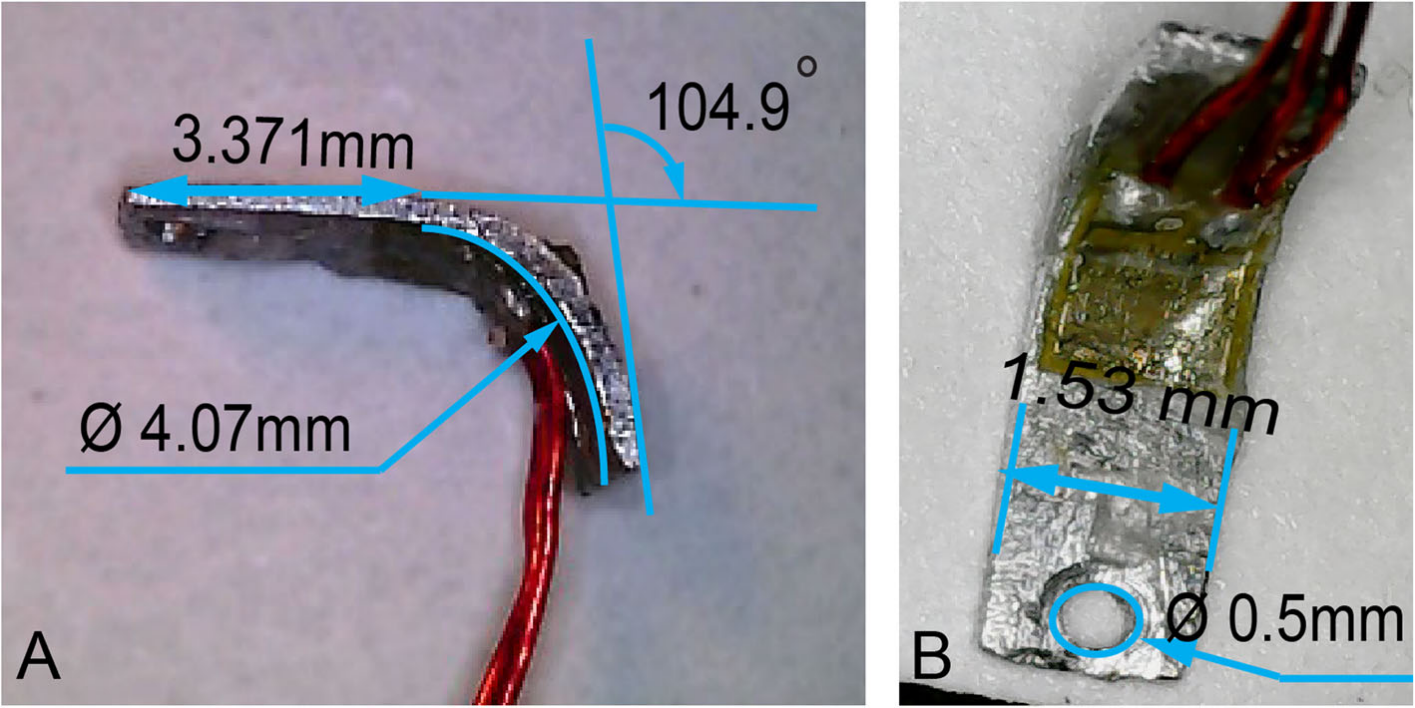
Sensing beam element with attached strain gauge; **A** side view orientation showing beam length, bend angle and radius of curvature, **B** front view orientation showing dimensions of width and mounting hole of the beam

The sensing element was fabricated using a 0.3*mm* thick aluminum foil cut using a shearing machine at the required dimensions established using FE analysis. A 0.5*mm* mounting through hole was drilled at the designed location. The start of the bend radius was marked according to the design. A dowel pin with 4*mm* diameter was used to achieve the designed bend radius for the aluminum strip at a desired bend angle of 100 degrees. The spring back effect of the material yielded a bend angle of 104.9 degrees. The fabricated beam element with an attached micro-strain gauge at the desired location is shown in Fig. 6. The assembled beam sensing element design parameters are measured using a Supereyes Microscope (Shenzhen, Guangdong, China) as shown in Fig. 6a and b.

The Form3 printer was used to prototype sensor components. The initial printing attempts were not successful due to the features and size of the components. The sensor head was printed in three different orientations and the sensor base in two different orientations in an attempt to fabricate defect free parts.

The print time needed to fabricate ten parts (six sensor head and four sensor base) was approximately 210 minutes. The parts from the 3D printer were then cleaned using Form Wash cleaning station with fresh isopropyl alcohol (approximate time needed 60 minutes).

**Fig. 7:**
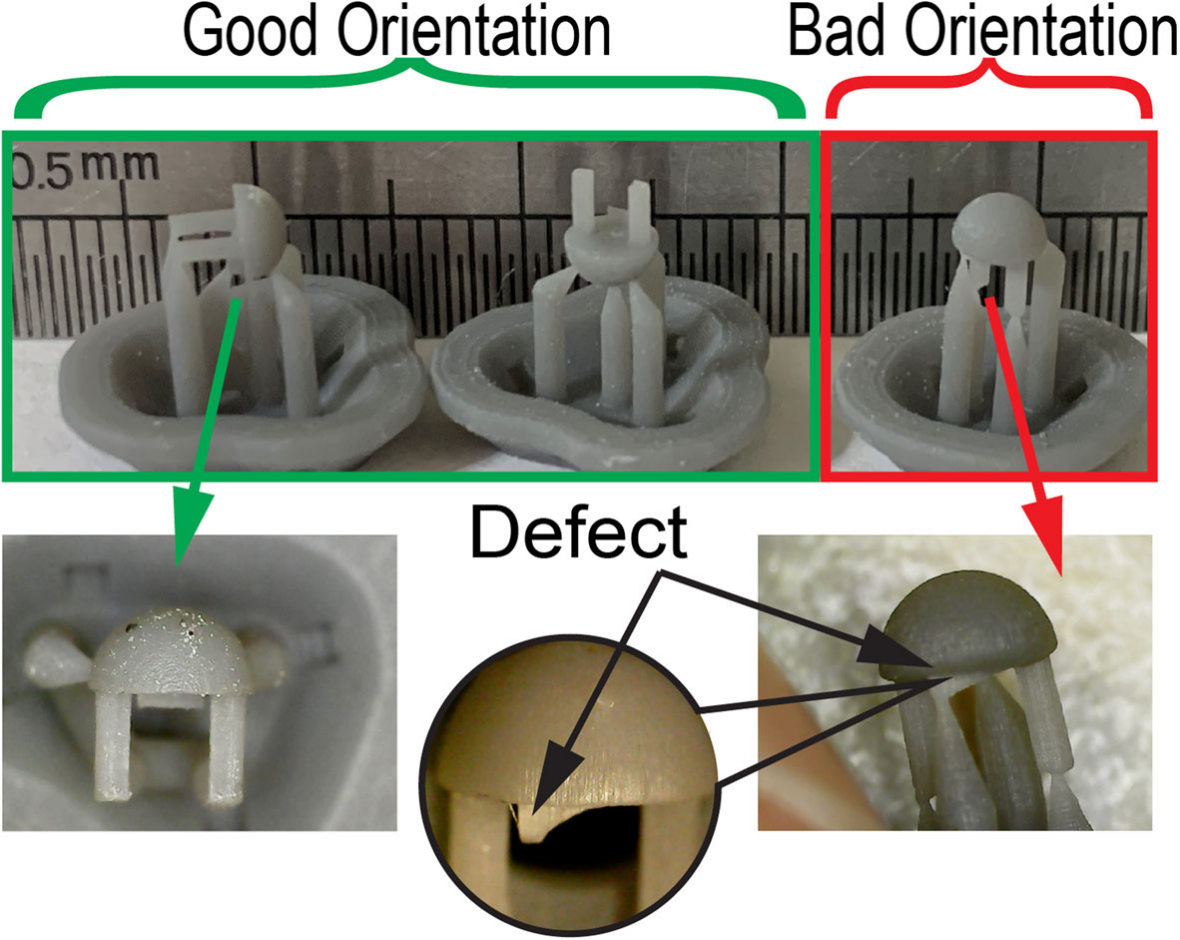
Actual scale cured sensor head component fabricated in different orientations to highlight good and bad orientations and defects

Figure 7 shows cured sensor head components in three different orientations. The build orientation of the components was defined considering the alignment of the finished part with respect to the print direction. The 3D printed sensor head components were classified according to their print orientation as either being good or bad.

Sensor head components printed in a bad orientation exhibited defects at the load transmitter feature as shown in Fig. 7. The support structure was provided only at one end of the load transmitter feature, where the other end had no support which lead to a failed part and thus a bad orientation. According to Formlabs fabrication specifications, the feature size (thickness) of an unsupported wall needed to be at least 0.2*mm* [38]. However, by printing the sensor head component with load transmitter feature thickness 0.2*mm* in three different orientations exemplified a limitation of the fabrication specification for wall thickness. The incomplete fabrication of the load transmitter feature in the upright position was addressed by re-orienting the sensor head in the vertical upside down and/or side orientations and successfully fabricated the component as shown in Fig. 7.

**Fig. 8:**
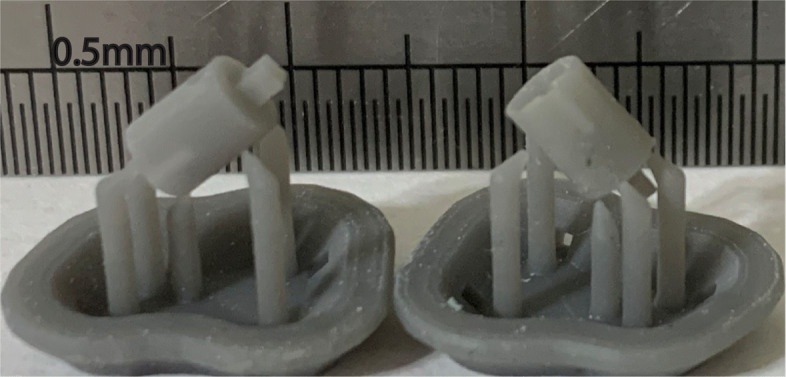
Actual scale cured sensor base component fabricated in different orientations

Figure 8 shows cured sensor base components in two different orientations. The sensor base is a hollow cylinder with embedded features and identifying the print orientation was important to ensure that no polymer was clogged inside the hollow portion of the component during fabrication which might cause issues during cleaning and curing. Both build orientations of the sensor base component were acceptable since this component had a hole feature at the bottom face and a rectangular open face on the top and either of them could act as the drain to avoid clogging up material.

**Fig. 9:**
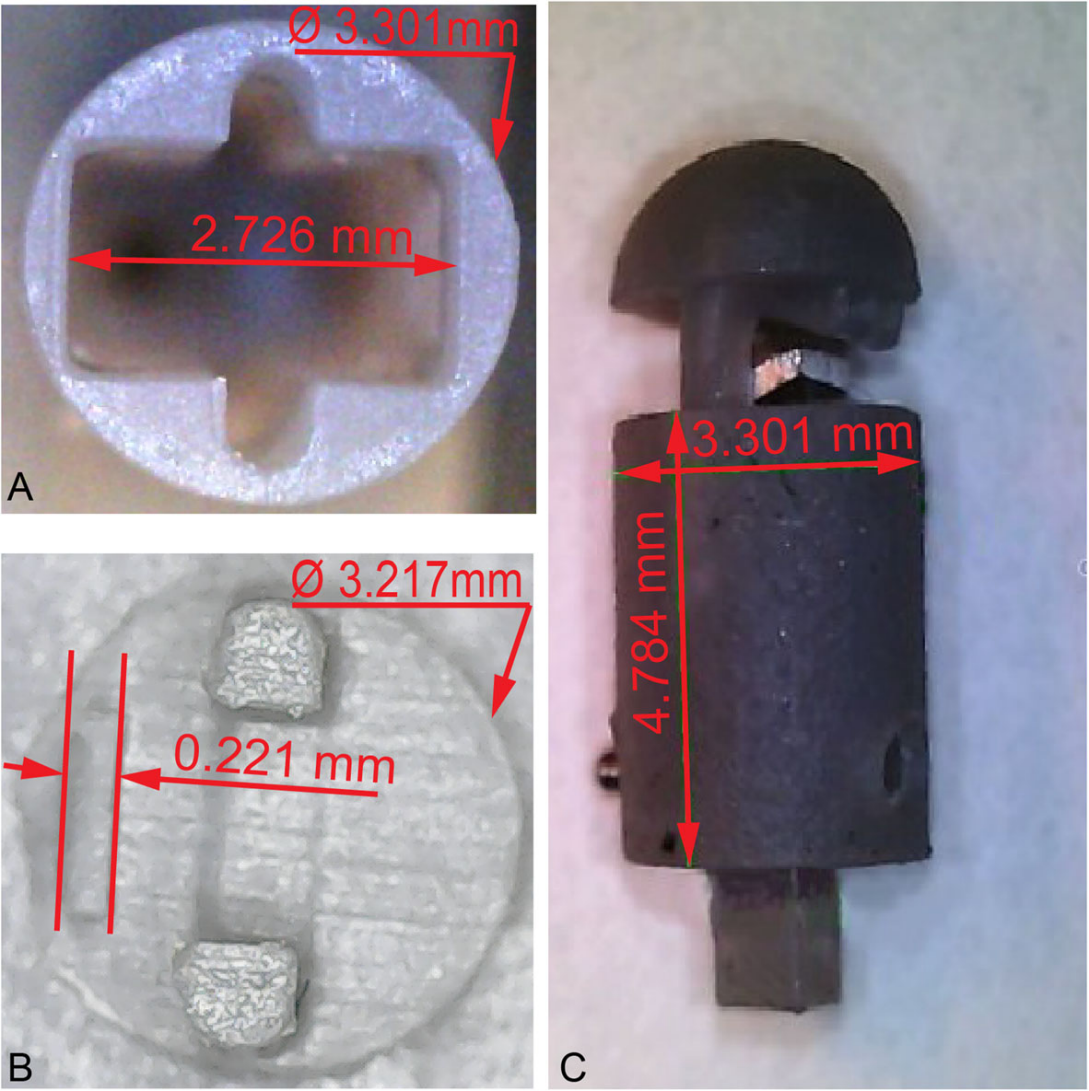
Cured sensor components fabricated using Form3 printer; **A** Top view of the cured sensor base component, **B** Bottom view of the cured sensor head component, **C** Assembled components with the beam element

Figure 9 shows the 3D printed and assembled sensor housing components with measurements using a Supereyes microscope. Figure 9a is the top view of the sensor base component, less than the desired sensor housing diameter of 3.5*mm*.

The rectangular slot in Fig. 9a was provided to accommodate the sensing element, where the semi-circular feature at either side of the rectangular slot were the guide ways for the legs of the sensor head component. Figure 9b shows the bottom view of the sensor head component. Figure 9c presents the prototyped sensor housing assembly with the sensor head, sensor base and beam components.

The defect free components obtained from the good orientation of the print are shown in Fig. 7 (sensor head) and Fig. 8 (sensor base). The components printed in the good orientation were not only defect free but also had good dimensional accuracy. The comparison of dimensions of the as designed and as fabricated components is presented in Table 2.

**Table 2:** Comparison of designed and fabricated dimensions of the 3D printed components of the sensor

Feature name	Designed	Fabricated^*^	Absolute
	dimensions (mm)	dimensions (mm)	Difference (*μm*)
Sensor base diameter	3.400	3.301	99
Sensor base height	4.650	4.784	134
Length of rectangular slot on sensor base	2.600	2.726	126
Sensor head diameter	3.400	3.217	183
Load transmitter thickness on sensor head	0.200	0.221	21

*measured using a Supereyes microscope system

### Sensor characterization

**Fig. 10:**
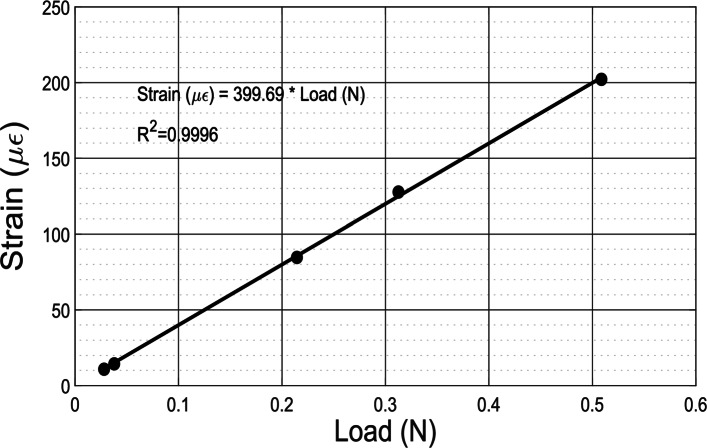
Evaluated strain from measured *ΔR*_*SG*_ as a function of applied load

The sensor characterization experiments were performed using dead weights corresponding to applied loads of 28*mN*, 37*mN*, 214*mN*, 311*mN*, and 509*mN* inclusive of the load holder weight of 18*mN*. Each load test was performed in five randomized trials for a total of 25 experiments. The analysis of the sensor characterization provided insight on the behavior of the micro-force sensor system. Figure 10 shows the calibration curve for the prototyped sensor in the force range of 0−509*mN*.

Each data point in Fig. 10 represents the calculated mean strain from the measured sensor signal for a particular load after all the randomized experimental trials were completed. The line represents the curve fit for the calculated strain as function of the applied load. The characterization test indicates excellent linear behavior (*R*^2^=0.999) between the strain gauge sensor output and applied load. According to the curve-fit equation (Fig. 10), the sensitivity of the sensor (strain/load) is evaluated to be 399.69*με*/*N*.

In the load range of interest, the resolution of the data acquisition device was 3*mΩ*, which corresponded to an equivalent load of 0.7*mN*. The sensor response from the preliminary characterization revealed that the sensor was capable of sensing an applied change in load equivalent to 18*mN* which was less than the 20*mN* desired resolution. This indicated the hardware resolution capability as being 29 times better than the desired sensor resolution.

The experimental measurements and theoretical results were used to develop an accuracy matrix for the performance of the sensor for a subset of the runs which is presented in Table 3. The first column represents the experimental run order number out of 25 experiments. The second column is the load applied on the sensor head. The third column is the calculated change in resistance in the strain gauge used to estimate the experimental strain according to Eq. 1. The theoretical strain for each loading case was obtained from the FE model at location *M* (refer Fig. 2).

**Table 3:** Accuracy matrix comparing experimental and FE theoretical results

Run	Applied	*ΔR*_*SG*_	Experimental	Theoretical	Error
Order	Load (mN)	(*Ω*)	Strain (*με*)	Strain (*με*)	(%)
3	28	-0.11	10.81	11.14	3.13
10	38	-0.15	14.73	14.65	2.12
1	214	-0.87	85.46	86.28	1.89
2	313	-1.28	125.73	125.91	1.40
4	509	-2.06	202.35	204.55	1.18

According to Table 3 the largest error occurs at the smaller applied load of 28*mN*. A precision of 99.9*%* was evaluated for each loading condition independently using Eq. 5.

**Fig. 11:**
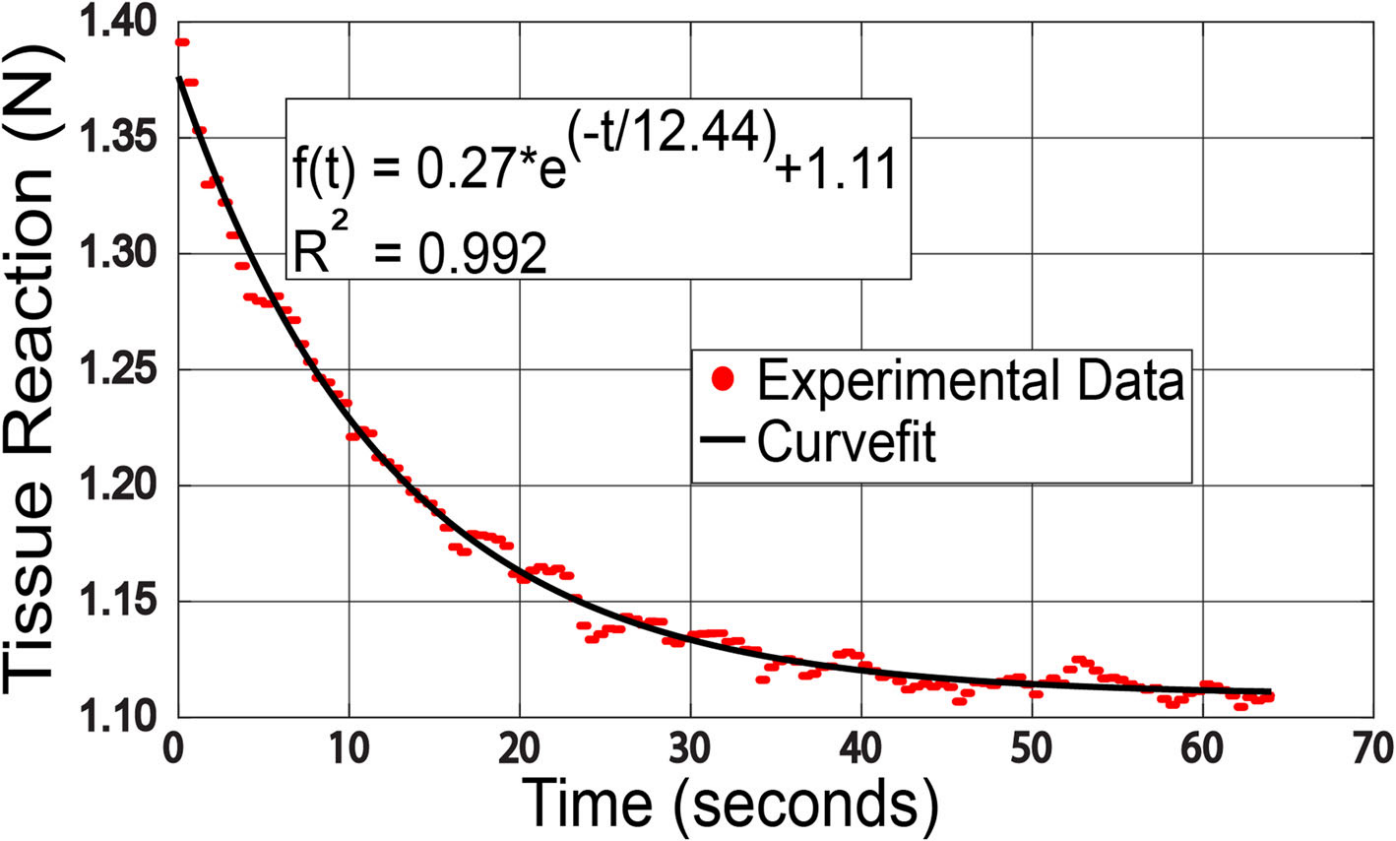
*In vivo* tissue response from the force sensor and curve fit

The in vivo operational performance of the sensor was evaluated using the developed testbed (see Fig. 4) on human forearm. After manually positioning the sensor at an indentation depth of 6.3*mm* using the micrometer dial, the forearm was held fixed and the tissue allowed to relax. The strain measured by the sensor due to this indentation depth corresponded to an equivalent load of 1.37*N*. The strain measurements from the sensor response as function of time were obtained using the same data acquisition setup used for the sensor characterization experiments. The strain measurements were post-processed according to Eq. 3 to obtain the relaxation force. The time data was also post-processed such that the relaxation state starts at time 0*sec*. The post-processed time series relaxation force data used for evaluation of biomechanical tissue properties is shown in Fig. 11.

The viscoelastic time constant of the Voigt model was evaluated by curve fitting the relaxation data set according to Eq. 6. The analysis estimated a viscoelastic time constant of 12.38*sec* with *R*^2^=0.992 with peak and residual forces of 1.37*N* and 1.11*N* respectively.

## Discussion

It has been demonstrated that the fabricated micro-force sensor using 3D printing technology was able to provide high performance and that this sensor could further be used to characterize biomechanical tissue property. The fabrication using the Form3 printer required a skilled operator to properly orient the parts during pre-processing in the PreForm software. Properly orienting the parts was an important step, to assure the features of the components were fabricated according to the specifications provided by Formlabs. If a skilled operator was not available, then the user was resorted to experiment with different orientations until an acceptable one was identified based on the features of the components. We experimented by placing sensor components to be fabricated using the Form3 printer at different orientations to identify the preferred orientation to obtain quality parts without defects.

The dimensions of the features of the 3D printed components were in good agreement with the designed geometric specifications. The maximum dimensional error evaluated for the fabricated components was 183*μm* for the sensor head diameter.

The performance of the sensor was evaluated using the assembled prototype. The characterization of the sensor performance using the developed testbed and dead weights demonstrated a linear behavior with a high regression coefficient. This indicated that the proposed sensor design and identified dimensions of the sensing element allowed the sensing element to operate in the recoverable elastic region of the material without experiencing plastic deformation.

The comparison of the theoretical strain obtained from the FE analysis and the experimentally measured strain showed a maximum accuracy error level of ∼3*%* with the error reducing as the applied load increased. The error behavior, i.e. error reduction as load increases, could be attributed to easier overcoming frictional losses due to the sliding of the sensor head legs in the sensor base slot. Even though the maximum error was ∼3*%*, further investigation to identify the factors that contribute to this error at the small load values is warranted.

The in vivo experiment was performed on a human forearm. The data in the relaxation region was post-processed and used to evaluate the viscoelastic time constant for the forearm according to the Voigt model of 12.38*sec*. The curve fit had a very high regression coefficient of 0.992. Even though this experiment was performed only on a single individual, the results of this experiment are encouraging. The sensor operation still needs to be verified by performing several experiments to evaluate the sensor performance matrix for different tissue types and different individuals.

The current operational testbed provides the user the tools to manually position the sensor to interact with the tissue. As such, the effect of the indentation strain rate on tissue characterization can not be evaluated. The lack of automated means to define the indentation strain rate is a limitation that needs to be addressed by improving the testbed. This improvement will allow the study of the indentation strain rate on tissue properties. The operational sensor performance needs to be further investigated in controlled in vivo experiments on animals or in clinical settings to characterize and compare viscoelastic properties of healthy and diseased tissue.

## Conclusions

In this paper, we presented the design, fabrication, assembly and characterization of a uniaxial miniature force sensor with design requirements of an overall diameter of less than 3.5*mm* and a load bearing capacity of 1.2*N*. The dimensions of the sensing element were identified using FE analysis considering performance requirements of resolution, maximum applied load, location of attached strain gauge for linear performance as well as size constraints.

3D printing provided several advantages compared to traditional machining processes such as rapid prototyping for miniaturized structures and features, fabrication of complex geometries and the ability to modify the design to meet the desired design specifications. Even though sensor components fabricated using the Form3 printer (inverted vat photopolymerization 3D printing platform) and Grey material exhibited good dimensional accuracy, load bearing capacity, function, and performance, extensive experimentation to identify the correct fabrication orientation to obtain acceptable defect free components had to be performed.

The overall sensing performance of the sensor was experimentally assessed using dead weights for its sensitivity, resolution, accuracy and precision. The sensitivity of the sensor was 399.69*με*/*N*. The designed sensor can sense reaction forces with a resolution of 18*mN*. The sensor exhibited good accuracy by estimating reaction forces with a 3.13*%* error. The sensor was also able to maintain high precision within 99% for all loading conditions.

The characterization of the sensor yielded a linear behavior with high regression coefficient. The characterized sensor was used for an in vivo experiment to evaluate operational performance using force relaxation data on a human forearm. The force relaxation data were used to find the viscoelastic time constant (*τ*=12.83*sec*) according to the Voigt model. In addition, the curve fit of the Voigt model equation for the relaxation data yielded a very high regression coefficient (*R*^2^=0.992).

The initial sensor performance was promising thus encouraging further investigation to improving the sensor design and performance and to employ it on animal studies and clinical setting. This type of micro-force sensor could be attached on delivery instruments to access confined spaces in the human body such as the bladder to interrogate tissue and use the measurements evaluate quantifiable viscoelastic tissue properties over extended time periods for medical diagnosis and care.

## Data Availability

The datasets generated and/or analyzed during the current study are available from the corresponding author upon reasonable request and not for commercial purposes.

## Abbreviations

UI: Urinary incontinence
IVP: Intravenous Pyelogram
POP: Pelvic Organ Prolapse
FBG: Fiber Brag Grating
3D: Three Dimensional
CAD: Computer Aided Design
UNM: Unified National Miniature
FE: Finite Element
SLA: Stereolithography
SLS: Selective Laser Sintering
FDM: Fused Deposition Modeling
3DP: Powder-Binding Bonding
LLM: Layer Laminate Manufacturing
LFS: Low Force Stereolithography
DAQ: Data Acquisition
